# CRISPR/Cas9 Delivery Potentials in Alzheimer’s Disease Management: A Mini Review

**DOI:** 10.3390/pharmaceutics12090801

**Published:** 2020-08-25

**Authors:** Amira Sayed Hanafy, Susanne Schoch, Alf Lamprecht

**Affiliations:** 1 Department of Pharmaceutics, Institute of Pharmacy, University of Bonn, 53121 Bonn, Germany; alf.lamprecht@uni-bonn.de; 2 Department of Pharmaceutics and Pharmaceutical Technology, Faculty of Pharmacy, Pharos University in Alexandria, Alexandria 21615, Egypt; 3 Department of Neuropathology, University of Bonn Medical Center, 53105 Bonn, Germany; susanne.schoch@uni-bonn.de

**Keywords:** CRISPR, Alzheimer’s disease, drug delivery, gene editing

## Abstract

Alzheimer’s disease (AD) is the most common dementia disorder. While genetic mutations account for only 1% of AD cases, sporadic AD resulting from a combination of genetic and risk factors constitutes >90% of the cases. Clustered Regularly Interspaced Short Palindromic Repeats (CRISPR)-associated protein (Cas9) is an impactful gene editing tool which identifies a targeted gene sequence, creating a double-stranded break followed by gene inactivation or correction. Although CRISPR/Cas9 can be utilized to irreversibly inactivate or correct faulty genes in AD, a safe and effective delivery system stands as a challenge against the translation of CRISPR therapeutics from bench to bedside. While viral vectors are efficient in CRISPR/Cas9 delivery, they might introduce fatal side effects and immune responses. As non-viral vectors offer a better safety profile, cost-effectiveness and versatility, they can be promising for the in vivo delivery of CRISPR/Cas9 therapeutics. In this minireview, we present an overview of viral and non-viral vector based CRISPR/Cas9 therapeutic strategies that are being evaluated on pre-clinical AD models. Other promising non-viral vectors that can be used for genome editing in AD, such as nanoparticles, nanoclews and microvesicles, are also discussed. Finally, we list the formulation and technical aspects that must be considered in order to develop a successful non-viral CRISPR/Cas9 delivery vehicle.

## 1. Introduction

Alzheimer’s disease (AD) is the most common type of dementia, affecting millions of people worldwide. Even though its initial discovery and description goes back 100 years, many open questions regarding the pathophysiology of the disease remain unanswered. The typical clinical profile of AD is characterized by memory loss and impaired cognitive functions, such as judgement, recognition, word finding and problem solving [1]. AD brains exhibit neuropathological alterations which represent the disease hallmarks: extracellularly accumulated β-amyloid (Aβ) plaques and intracellular neurofibrillary tangles (NFTs) comprising hyperphosphorylated tau protein [2].

The presentation of AD symptoms between the ages of 30 and 65 years is classified as “early-onset” AD, which is mainly genetic for 92–100% of cases [3]. On the other hand, “late-onset” AD symptoms start to appear beyond the age of 65 years. In the United States alone, around 5.8 million people suffer from AD, according to a 2019 report, 45% of which fall into the age group of 75–84 years [4]. In 2050, the number of AD patients is expected to rise to 14 million in the US alone [2]. It is widely thought that external factors beyond the genetic predisposition might be responsible for disease presentation.

**Figure 1:**
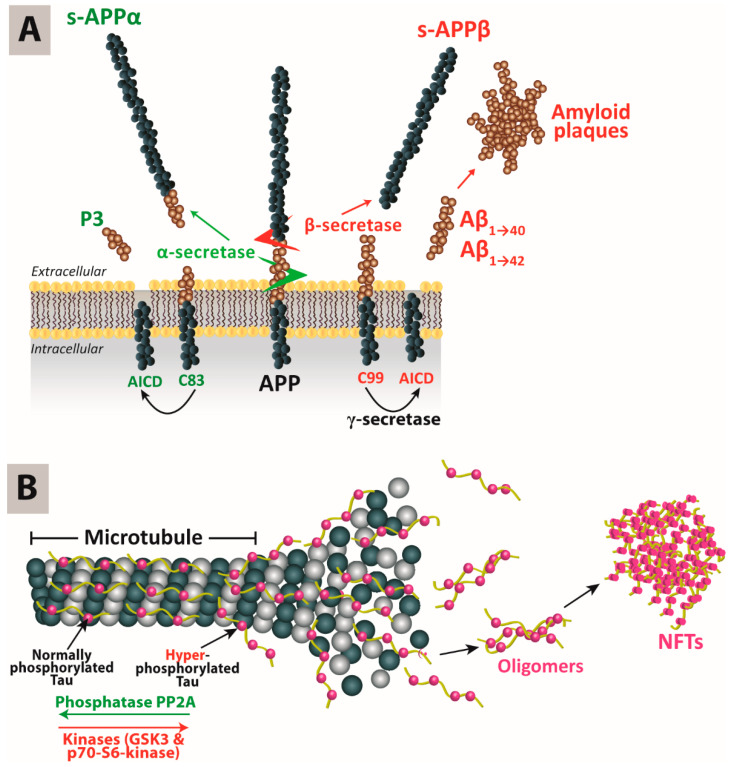
The pathogenesis hypotheses that have been proposed to explain the most common hallmarks of Alzheimer’s disease. (**A**), Amyloid cascade hypothesis. (**B**), Tau hypothesis. AICD, Amyloid precursor protein Intracellular C-terminal Domain; APP, amyloid precursor protein; Aβ, beta-amyloid protein; GSK3, Glycogen Synthase Kinase 3; NFTs, neurofibrillary tangles. Reprinted with minor modification from Wen et al., Journal of Controlled Release, published by Elsevier, 2019 [9].

The accumulation of Aβ in the brain is explained by the amyloid cascade hypothesis: Amyloid-precursor protein (APP) is a transmembrane protein that undergoes proteolysis under the concerted actions of α-, β- and γ-secretases. AD is associated with an increased activity of the β-secretase 1 (BACE1), leading to the accumulation of Aβ monomers into oligomers and subsequently Aβ plaques. Cleavage of APP by the β-secretase results in the formation of the C99 fragment, which in turn is cleaved by the γ-secretase, of which presenilin (*PSNE1/2*) is one component, at positions 40 or 42 to produce Aβ monomers Aβ_40_ and Aβ_42_. On the other hand, the α-protease can also cleave APP at a different site that minimizes the production of β-amyloid monomers (Figure 1A) [5].

The formation of NFTs in AD brains is explained by the tau hypothesis. Tau (tubulin-associated unit) is an essential protein for the formation and stabilization of the microtubule cytoskeleton [6]. Among the six tau isoforms, 3-repeat (3R) and 4-repeat (4R) are primarily expressed in the neuronal axons of adult human brains. Tau is the target of multiple kinases and phosphatases. In AD, it has been proposed that 3R and 4R tau might accumulate in a hyperphosphorylated form, resulting in NFTs or threads if present within neuronal cell bodies or axons, respectively. That cascade of events might precipitate tau pathology. Recently, it has also been suggested that tau oligomers might be the microstructures mediating neuropathology (Figure 1B) [7]. It has been reported that there could be a molecular link between NFT formation and Aβ deposition. The latter has been found to initiate reduced neuroplasticity, neuronal viability and microtubule disassembly, and also inhibit the transport of mitochondria along microtubules. Therefore, it has been hypothesized that tau neurotoxicity could be an event downstream from Aβ accumulation [8]. However, this hypothesis still needs to be experimentally verified.

## 2. Familial and Sporadic Alzheimer’s Disease

AD can be categorized into familial (FAD) and sporadic (SAD). FAD runs in certain families and is responsible for only 1% of AD cases. It has been well reported that FAD is primarily correlated with genetic factors affecting Aβ metabolism. Currently, three different genes are thought to be involved in at least 50% of FAD cases, namely *APP*, presenilin-1 (*PSEN1*) and presenilin-2 (*PSEN2*) [3]. The mutations found in those genes lead mostly to an abnormal Aβ production, aggregation or clearance, i.e., Aβ metabolism. So far, more than 400 mutations have been reported in *APP*, *PSEN1* and *PSEN2* (according to the database available on http://www.alzgene.org/).

In the *APP* gene, the majority of mutations are in proximity to the α-, β- or γ-secretase cleavage sites, explaining the link between the mutations and the altered Aβ metabolism. In general, an increased production of Aβ_42_ fragments, which are neurotoxic, can initiate a series of neuroinflammatory reactions leading to an aggravated deterioration in brain cognitive functions. It has been suggested that the functional impact of mutations in the *PSEN1* and *PSEN2* genes is an increase in γ-secretase activity, as well as an elevation of Aβ_42_ generation, thereby shifting the Aβ_42_/Aβ_40_ ratio and thus ultimately altering APP processing by γ-secretase [10].

On the contrary, SAD accounts for more than 90% of AD cases, and the underlying causes are less well understood than for FAD. SAD is reported to be 70% predisposed by genetic variants and 30% by other risk factors. The latter includes non-modifiable factors such as ageing [11], gender [12] and hormones [13], and modifiable factors including physical activity, social standards, education, cardiovascular health, obesity, stress [14] and others [15,16].

In addition, Apolipoprotein E (*APOE*), which plays a crucial role in neuroinflammation and neuroplasticity, is strongly associated with SAD. *APOE* has three common alleles, namely ε2, ε3 and ε4. The existence of an ε4 allele at the *APOE* locus is a well-reported risk factor for SAD precipitation. It has been found that heterozygous *APOE* ε4 carriers are two to three times more prone to developing AD. Families having homozygotes of *APOE* ε4 have a 10–15 times increased AD risk. However, the possession of the ε4 allele does not necessarily lead to AD development; it only increases the risk [17]. Furthermore, alteration in cholesterol metabolism, for example in hypercholesterolemia and hypertension, has been associated with a predisposition for SAD. Cholesterol itself cannot cross the blood–brain barrier (BBB). However, the oxidized forms of cholesterol (oxysterols) can adequately cross the BBB. It has been found that increased cholesterol levels are accompanied by increased 27-hydroxycholesterol production and the latter’s influx from blood to brain [18]. Hyperphosphorylated tau or Aβ accumulation were found to be correlated with 27-hydroxycholesterol increased brain and cerebrospinal fluid concentrations [19].

## 3. CRISPR/Cas9

Clustered Regularly Interspaced Short Palindromic Repeats (CRISPR)-associated protein (Cas9) constitutes a recently developed powerful genome editing tool that has the potential to treat diseases for which treatments are still lacking or ineffective. After its initial identification in 1987 by Ishino [20], subsequent studies have shown that the CRISPR/Cas9 system represents one part of the immune system in bacteria, protecting them from the unwanted integration of mobile genetic elements, like viruses or plasmids. It has been adapted to the laboratory setting to explore its potentials, as pioneered by Doudna and Charpentier [21]. In recent years, the CRISPR/Cas9 system has been studied in great detail and has been further improved, for example by minimizing the off-target effects and editing efficiency, and has been widely used both in basic research and in translational approaches [21,22].

**Figure 2:**
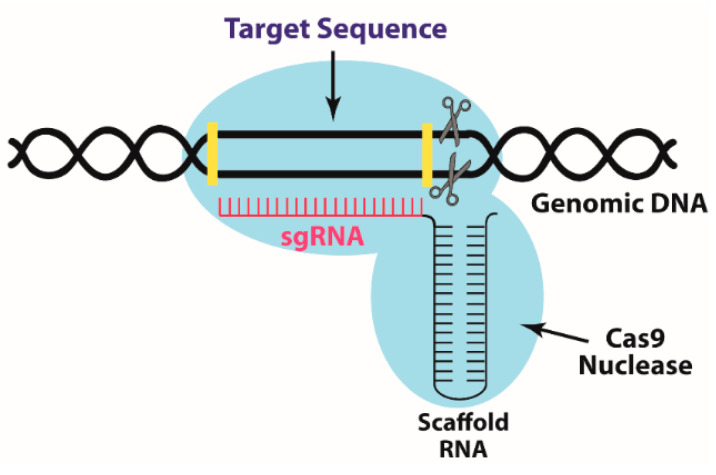
A schematic representation of CRISPR/Cas9 system.

The CRISPR/Cas9 system consists of two main components: a single-guide RNA (sgRNA) and Cas9 enzyme. The sgRNA recognizes the targeted DNA sequence, whereby in the design process several parameters have to be considered to improve specificity, while the Cas9 protein is an endonuclease that acts as scissors to cut the DNA double strands (Figure 2). There are different types of CRISPR/Cas systems, which can be divided into Class 1 (types I, III, IV) and Class 2 (types II, V, VI). Class 1 comprises several Cas proteins working together, while Class 2 systems use a single Cas protein, thus they are simpler and preferable in genome editing [23]. Amongst Class 2, the type II CRISPR/Cas9 is the most extensively studied and used system.

Upon recognizing the target genomic sequence, the Cas9 protein creates a double-stranded break. Thereafter, two pathways can be initiated in order to repair this break: non-homologous end joining (NHEJ) or homology directed repair (HDR). NHEJ pathway results in insertions and deletions (InDel), which lead to DNA frameshifts and/or premature stop codons and thereby result in gene inactivation. On the other hand, the HDR pathway helps replace the faulty/mutated sequence with a correct one. In order to initiate HDR, the correct DNA sequence is inserted into the targeted position with the help of a donor DNA template [24]. NHEJ can occur in all phases of the cell cycle, whereas HDR is restricted to the S or G phase. In general, the HDR pathway is the more reliable DNA repair mechanism, although it is less efficient than the NHEJ pathway.

**Figure 3:**
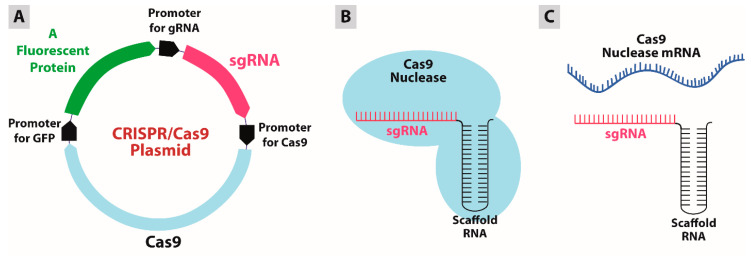
Strategies for genome editing using the CRISPR/Cas9 technology. (**A**) plasmid-borne CRISPR/Cas9 system. (**B**) Cas9/sgRNA complex. (**C**) Cas9 mRNA and sgRNA mixture.

In order to edit a target gene using the CRISPR/Cas9 system, there are three potential ways to apply the system: plasmid-borne CRISPR/Cas9 system, purified Cas9/sgRNA complexes, or a mixture of Cas9 mRNA and sgRNA (Figure 3). Each strategy has its own advantages and disadvantages, as summarized in Table 1.

## 4. Applications of CRISPR/Cas9 in the Treatment of Alzheimer’s Disease

As only 1% of AD cases are familial, i.e., caused by genetic mutations, it seems that genome editing by CRISPR/Cas9 would not be beneficial in SAD. However, it is well established that both FAD and SAD involve an altered Aβ metabolism. Therefore, correcting the increased Aβ production could be a therapeutic approach regardless of the genetic background. Table 2 provides an overview of studies that have applied the CRISPR/Cas9 technology in therapeutic strategies for experimental models of AD (sporadic or familial).

Treatment of FAD with genome editing can target the mutations in three genes, *APP*, *PSEN1* and *PSEN2*, as well as interfere with Aβ production. In a first proof of concept study, György et al. investigated the therapeutic potential of CRISPR/Cas to decrease the pathogenic Aβ concentration by selectively disrupting the so-called Swedish mutation, *KM670/671NL APP* (APP^swe^), which is a mutation in the *APP* gene located at the β-secretase cleavage site [25]. The mutation causes hyperactivity of the β-secretase enzyme resulting in elevated Aβ brain levels. Viral vectors containing the sgRNA targeting the APP^swe^ allele and the Cas9 enzyme were injected into the hippocampus of an AD mouse model expressing the APP^swe^ mutation (*Tg2576* mouse line). DNA sequencing one month after the injection revealed about 2% InDels in the APP^swe^ allele. When considering the efficiency of genome editing, it has to be taken into consideration that there are about 100 copies of the transgene per neuron in the transgenic *Tg2576* mice [25]. Further studies are needed in order to assess the extent of allele disruption needed for AD progression to disrupt or correct the mutated alleles before the appearance of symptoms.

Recently, CRISPR/Cas9 was applied to target *BACE1* in two mouse models: *5xFAD* mice (expressing human *APP* and *PSEN1* transgenes with a total of five AD-linked mutations) and *App* knock-in mice [27]. In this study, the negatively charged Cas9/sgRNA complex specific to the BACE1 gene was complexed with the R7L10 peptide to form nanocomplexes that were around 100 nm in diameter. The nanocomplexes were directly injected into the hippocampus. Genome sequencing and Aβ quantification were performed 8 and 12 weeks later. Sanger sequencing revealed about 70% lower BACE1 expression in the hippocampi of treated mice. In addition, a significant reduction in Aβ levels and in cognitive deficits was observed in mice injected with Cas9 nanocomplexes compared to control mice. Moreover, the authors investigated the possible off-target effects caused by nanocomplexes. They showed that mutations in both treated and control mice did occur, although the extent of such effects was low [27]. This study showed that application of the CRISPR/Cas9 system using non-viral Cas9 nanocomplexes constitutes a potential general therapeutic approach. However, this delivery method does not allow for the widespread targeting of neuronal circuits, and therefore most likely cannot stop the progression of AD.

Moreover, CRISPR/Cas9 can be used to target the C-terminus of APP without affecting the N-terminus, which has been suggested to have important physiological roles. Editing the C-terminus interferes with the APP internalization and initiation of the amyloidogenic pathway. The developed lentiviral CRISPR/Cas9 was tested in HEK293 and neuro2a cells, as well as being injected into the dentate gyrus of healthy *C57BL/6* mice [30]. While this study provides a proof of concept that silencing *APP* selectively decreases BACE1 activity without detectable off-target effects, investigations are needed in order to verify the efficiency and long-term effects of silencing BACE1 in AD models.

The development of therapeutic genome editing strategies for SAD has focused on targeting *APOE* ε4. In one study, a novel innovative “base editing” approach was developed and applied to correct a disease-relevant mutation in the *APOE* gene [31], involving an irreversible direct conversion of a single targeted base to another in order to convert *APOE4* into *APOE3r*, as the latter confers a lower AD risk. While CRISPR/Cas9 systems induce double-stranded DNA breaks, in this system CRISPR/Cas9 was fused with a cytidine deaminase enzyme to retain sgRNA programmability but to not introduce double-stranded DNA breaks, thus minimizing the side effects resulting from unwanted mutations. The developed ‘base editing’ system resulted in a 15–75% permanent correction of DNA with <1% InDels when tested in HEK293T cells and immortalized mouse astrocytes containing the *APOE* ε4 isoform of the *APOE* gene [31].

Even though the APOE ε3 and ε4 alleles differ only in one nucleotide, the APOE ε4 allele could be selectively targeted using CRISPR/Cas9 after lentiviral delivery. It was found that APOE ε4 protein levels decreased by 56%, without affecting APOE ε3, in mouse astrocytic cells expressing both human alleles [32].

Furthermore, it has been reported that SORL1, the gene encoding the protein SORLA, is linked to early-onset and late-onset AD. SORLA is a sorting receptor that exists in almost every CNS cell and plays crucial roles in the regulation of APP processing. The expression of SORLA significantly declines in SAD [33]. In a recent study by Knupp et al. [34], CRISPR/Cas9 was used to generate *SORL1*-deficient human induced pluripotent stem cell (hiPSC) lines. They measured the endosomal size in neurons and microglia differentiated from this cell line. It was found that the loss of SORL1 led to endosomal enlargement in neuros, but not in microglia. Moreover, APP localization within the endosomal network was altered as a result of *SORL1* deficiency [34]. This study could help in understanding the side cytopathological pathways involved in Aβ deposition.

## 5. Delivery of CRISPR/Cas9 in Alzheimer’s Disease

As detailed above, the CRISPR/Cas9 methodology shows promise for the development of novel therapeutic approaches for the treatment of AD. However, its effective, safe and efficient delivery remains a challenge that needs to be tackled in order to translate this genome editing technology into real-life applications. Generally, the CRISPR/Cas9 system can be delivered via viral or non-viral approaches. The selection of a suitable delivery vehicle depends on the employed CRISPR strategy, and whether in vitro and/or in vivo delivery are intended. For example, the Cas9/sgRNA complex and oligonucleotides are negatively charged, while the Cas9 protein is positively charged [35]. In the following sections, an overview of both approaches is given with a particular focus on previous studies aiming at the amelioration of AD pathological conditions.

## 6. Viral Vectors

Using viral vectors is a classical approach that has been previously used to deliver CRISPR/Cas9 in vitro and in vivo because of their efficiency and long-term stability. The most widely used viruses are the adeno-associated virus (AAV) and lentivirus. By far, viral vectors are the most efficient delivery systems of plasmid-based CRISPR/Cas9. However, they can introduce unintended mutations with serious side effects. In addition, they can lead to severe immune responses that can be fatal.

AAV is the most commonly used viral vector due to its high infection ability, mild immunogenicity [36], and the fact that they do not normally integrate into the human genome [37]. It is able to infect cells with little to no immune reactions. The AAV genome consists of a single-stranded DNA, with >200 variants [38]. One study reported on using two separate AAV vectors packaging APP^sw^-specific gRNA and Cas9 targeting the AD-causing *KM670/671NL APP* mutation.

The viruses were tested in vitro in primary neuronal cells from *Tg2576* mice embryos and in vivo via intrahippocampal injection in *Tg2576* mice. This treatment led to a 60% reduction in Aβ production in the human-derived fibroblasts [25]. Compared to AAV, lentivirus is more difficult to purify in large quantities, and is more likely to provoke immune reactions and integrate into the human genome at high efficiency [37]. As AAV has a lower packaging capacity of only 4.7 kb, the co-injection of two viruses might be necessary, which complicates the process as both might not infect the same cell simultaneously. However, longer DNA inserts (8–10 kb) can be incorporated into lentivirus, but with a lower brain spreading efficiency [39]. As indicated in Table 2, lentivirus has been used to target three different genes in FAD and SAD, which are *APP* [30], *APOE ε4* [32] and caspase-6 [40].

## 7. Non-Viral Vectors

While viral vectors are generally considered more efficient in delivering CRISPR/Cas9 to cells, non-viral vectors offer higher safety, better cost-effectiveness, and versatility in terms of the size of the transgenic component. Thus, non-viral vectors are considered more suitable for applications in AD. Although various non-viral vectors are available, the selection of which vector to use depends primarily on the type of CRISPR/Cas9 tool. Because of the large size of plasmid-borne CRISPR/Cas9, only some non-viral vectors might be appropriate for the former’s delivery.

Nanocomplexes can be simply prepared by complexing the negatively charged nucleic acid cargo, in this case CRISPR/Cas9, with positively charged peptides. They are known to be less immunogenic compared to viral vectors. As they can be functionalized with ligands, they would serve several applications. However, delivering nanocomplexes to the brain is challenging, as they cannot cross the BBB efficiently via the systemic route, and they get actively removed from the blood circulation by the reticuloendothelial system (RES). Therefore, intrathecal and intracerebroventricular injections are typically used. Direct injection methods, however, require multiple injections to achieve proper distribution across the brain, limiting their applicability. Park et al. [27] prepared nanocomplexes composed of R7L10 peptide complexed with Cas9-sgRNA ribonucleoprotein targeting the BACE1 gene. The nanocomplexes were directly injected into the hippocampi of *5xFAD* and *App* knock-in AD transgenic mice. They reported that the nanocomplexes successfully targeted the BACE1 gene, attenuating its expression without a significant off-target mutation rate in vivo. Moreover, the nanocomplexes were able to improve cognitive dysfunction in *5xFAD* transgenic AD mice. Interestingly, the study by Park et al. [27] is the only one that has reported on the in vivo use of non-viral vectors for CRISPR/Cas9 gene editing employing AD models. Intrahippocampal injection, selected as the route of administration in this study, delivers the nanocomplexes directly into the site of action. However, applying this technique to human subjects faces some challenges, as the procedure is invasive, requires deep anesthesia, holds infection risk, restricts the volumes that can be injected, and is inapplicable to repetitive administrations.

Apart from CRISPR/Cas9 delivery, other delivery vehicles carrying siRNA have been developed to target AD across the BBB. Polymeric nanocomplexes composed of poly(mannitol-co-PEI) gene transporter (PMT) modified with rabies virus glycoprotein (RVG) have previously been reported on in 2015 [39]. The polymer was complexed to siRNA against BACE1. The nanocomplexes were proposed to have an enhanced gene delivery capability due to the RVG ligand, which promotes crossing the BBB and targeting neuronal cells. A noncontact co-culture of capillary endothelial cells from mouse brains (bEnd.3) and rat astrocytoma cells (B-23) was used as an in vitro BBB model to study the BBB’s permeability to nanocomplexes. It was found that the latter’s permeability was 2.2-fold higher than control nanocomplexes, which were prepared similarly but without incorporating the RVG ligand. It was suggested that the internalization of RVG nanocomplexes was facilitated by RVG binding to nicotinic acetylcholine receptors. The prepared nanocomplexes transfected Neuro2a cells with high efficacy and downregulated BACE1 expression. The PEGylation of nanocomplexes was essential to increase their circulation time, and reduced the risk of having them identified by the reticulo-endothelial system (RES). However, PEG decreases transfection efficiency as it generates a positively-charged shield hindering attachment to cell membranes. It was suggested that the RVG ligand overcomes this PEG hindrance, thereby improving the cellular uptake of nanocomplexes. The in vivo study, performed on BALB/c mice via intravenous injections of the nanocomplexes, lead to the silencing of BACE1 by 2.32- and 3.03-fold in the cortex and the hippocampus, respectively. The silencing capability of nanocomplexes was further verified by the reduction of Aβ_1–42_ levels in the brain cortices. Regarding toxicity, hepatic and renal functions were maintained, and no induction of inflammatory cytokines or anti-peptide antibodies was reported. The reported nanocomplexes represent a promising delivery system for siRNA therapeutics, and could be extrapolated to deliver CRISPR/Cas9 therapeutics as well. Utilizing the intravenous route of administration is advantageous, as it allows the injection of larger volumes with an absolute bioavailability without the need for sophisticated technical abilities. However, an unknown body distribution may lead to a significant loss of therapeutic potential, since the efficacy of such a delivery system should be investigated in AD models especially because AD affects the BBB permeability, and could potentially impact the targeting and efficacy of nanocomplexes.

In the following, we present further non-viral delivery vehicles that have been used previously for CRISPR therapeutics delivery in pathologies other than AD. The following delivery systems could be promising for applications in AD.

DNA nanoclews can be a potential approach for delivering the Cas9/sgRNA complex. The traditional assembly of DNA nanostructures is based on base-pairing, which is complicated and time-consuming. On the contrary, DNA nanoclews, first reported on by Sun et al. [35], are nanosized DNA cages that contain polyethylenimine to exert a positive charge for better endosomal escape and cell uptake. They offer a greater stability due to the increased charge density. Such nanoclews are prepared by rolling circle amplification. Nanoclews carrying sgRNA/Cas9 complex-targeting enhanced green fluorescent protein (EGFP) were locally injected into the tumors of EGFP tumor-bearing mice, and manifested about a 25% decreased expression of EGFP 10 days post-treatment [35]. This study has paved the road for the application of nanoclews in AD, although local injection stands as a challenge. Besides their advantages, nanoclews might induce immunogenic reactions that still require further investigation.

In addition, lipid nanoparticles and polymeric nanoparticles have potential as CRISPR/Cas9 delivery tools as well. They have been extensively employed before to deliver gene editing cargos in cancer [41], hepatitis and other viral conditions [42]. However, their possible application in AD management remains to be investigated.

Mout et al. [34] prepared nanoassemblies formed by the mixing of arginine-functionalized gold nanoparticles and the Cas9/sgRNA complex-targeting human AAPS1 gene. As the nanoparticles are positively charged, a glutamate peptide tag was inserted at the N-terminus of the Cas9 protein, providing negative charge. To enhance nuclear targeting, a nuclear localization signal was introduced into the Cas9 C-terminus. The nanoassemblies were instantaneously able to fuse with cell membranes and target the nuclei in a few minutes via a cholesterol-dependent membrane process, rather than cellular endocytosis. Therefore, they accomplished a delivery efficiency of about 90% in different cell lines, with a 30% gene silencing efficiency [34]. It is worth noting that this study provided only a preliminary data set in cell culture, which does not necessarily reflect the nanoparticles’ targeting efficiency in vivo.

Gold nanoparticles have also been utilized in a study conducted by Lee et al. [43]. They developed “CRISPR-Gold”, which is a complex delivery vehicle comprised of gold nanoparticles conjugated with DNA, and complexed with donor DNA, Cas9-sgRNA and cationic poly(N-(N-(2-aminoethyl)-2-aminoethyl) aspartamide) (PAsp(DET)). The latter acts as an endosomal disruptive polymer, which facilitates cellular uptake via endocytosis and triggers endosomal disruption, freeing CRISPR-Gold into the cytoplasm. Then, the cytoplasmic glutathione assists the release of donor DNA and Cas9-sgRNA. CRISPR-Gold targeting the CXCR4 gene achieved 3–4% HDR efficiency in various human cell types, such as embryonic stem cells and primary bone-marrow-derived dendritic cells, in addition to a comparable efficiency in primary myoblasts from *mdx* mice, which supports the application of this non-viral delivery vehicle in a plethora of genetic pathologies. A single local injection of CRISPR-Gold into the gastrocnemius and tibialis anterior muscle in *mdx* mice resulted in the correction of the mutated dystrophin gene responsible for the congenital Duchenne muscular dystrophy [43]. Moreover, the inflammatory cytokine profile did not significantly change after CRISPR-Gold injection, indicating the latter’s tolerability and low toxicity. As CRISPR-Gold was locally injected into the affected muscle, it would be impossible to estimate its targeting efficiency when injecting it intravascularly to manage AD.

Recently, attention has been drawn to the application of microvesicles for the delivery of CRISPR/Cas9 therapeutics. These are extracellular vesicles 100–1000 nm in diameter. Via the budding of cell membranes, microvesicles are formed and shed into the medium. Generally, a ‘producer’ cell line is transfected with sgRNA, Cas9 protein and a microvesicle-inducing protein (such as RAB proteins) [44]. The cells produce microvesicles containing the Cas9-sgRNA complex, which get shed into the medium and are subsequently purified and re-used to deliver their gene-editing cargo to the targeted cells.

Gesicles are microvesicles produced via overexpression of the glycoprotein vesicular stomatitis virus G (VSV-G). A study reported on delivering Cas9-sgRNA to inactivate HIV proviral activity in the CHME-5 microglial cell line using gesicles [45]. The authors preferred to deliver Cas9 protein in a complexed form so as to limit the duration of Cas9 activity, in order to avoid developing HIV strains resistant to CRISPR/Cas9 and obtain less unwanted off-target mutations [37]. The gesicles induced mutations of the promoter and the excision of the HIV provirus, leading to diminished proviral activity. Moreover, the delivery of gesicles did not affect cell viability. Despite the shortage of in vivo studies, microvesicles might be a promising non-viral delivery vehicle for CRISPR/Cas9 therapeutics in AD management.

Overall, studies that involved the brain delivery of CRISPR/Cas9 therapeutics, or siRNA, to manage AD adopted either local or intravascular routes of administration, given the respective advantages of each. Even though oral delivery is convenient, patient-friendly and non-invasive, it is extremely challenging due to the multiple barriers that the delivery system has to cross in order to deliver its gene editing cargo to the blood [12]. On the other hand, the intranasal route has been getting more attention as it is believed to allow the bypassing of the BBB non-invasively and rapidly [13], suggesting nose-to-brain delivery as one way to quickly push CRISPR/Cas9 therapeutics in AD into clinical study. Finally, intraperitoneal and subcutaneous injections are alternative routes that could be exploited, but taking into consideration their disadvantages concerning pharmacokinetics, they would probably only be a fallback option.

## 8. Concluding Remarks, Challenges and Future Aspects

CRISPR/Cas9 is a promising gene editing tool that implies multiple potentials of treating AD, the most common dementia in the elderly population worldwide. An altered Aβ metabolism is commonly found in FAD and SAD, regardless of the genetic factors. Therefore, correcting increased Aβ production using CRISPR/Cas9 technology can be an effective therapeutic strategy. Additionally, CRISPR/Cas9 can be utilized to correct mutations in *APP*, *PSEN1* and *PSEN2*, which are commonly mutated in FAD.

A successful non-viral brain delivery of the CRISPR/Cas9 tool to manage AD is faced with a number of challenges. Ideally, the formulated vector should be stable and efficiently deliver the cargo to the site of interest. Upon reaching the targeted cells, the vector should be internalized, escape lysosomal degradation and target the nucleus. The preceding series of barriers can get more complicated in the case of CRISPR/Cas9 plasmids.

It is important to take the large size of CRISPR/Cas9 into consideration before designing the formula. The Cas9/sgRNA complex is preferred over plasmid-assisted delivery approaches due to the former’s smaller size. Furthermore, the components of these formulations are all liable to degradation by circulating nucleases and proteases. Although it is extensively used to minimize the identification of these systems by the RES, PEGylation concomitantly decreases cellular uptake and could generate specific PEG-antibodies, resulting in immunogenic responses [46]. As non-viral vectors are generally preferred over viral ones for in vivo applications, optimizing the different formulation aspects is the bottleneck limiting real-life translatability.

For the route of administration, the systemic route is the most studied due to its in vivo feasibility, especially for AD patients, although it is more challenging in terms of the stability and targetability of the delivery vector. Therefore, intrathecal and intracerebroventricular injections are typically used. Stereotaxic microinjection surgery has already been reported to deliver gene therapeutics to Parkinson’s disease brains in vivo [47]. This procedure might be challenging in AD due to the widespread nature of the amyloid pathology. Moreover, the intranasal route could also be a promising approach to bypassing the BBB, yet further clinical studies need to be conducted on the nasal delivery of CRISPR/Cas9 therapeutics.

As genome editing is an irreversible process, further research is a must in order to ensure the safety of CRISPR/Cas9 treatment. Studies investigating the long-term effects of CRISPR treatment and conceivable off-targets are still lacking. Any application in humans must first be scrutinized under strong ethical considerations. One of the advantages of CRISPR/Cas9 gene editing is that it is somatic rather than germline. Thus, the gene editing results will manifest only in the treated individual and will not be passed to future generations [12].

While CRISPR/Cas9 involves double-stranded DNA breaks, prime editing has recently been developed so as to correct gene mutations without inducing double-stranded breaks accompanied by unwanted off-targets. Instead, prime editing uses a catalytically impaired Cas9 fused to a reverse transcriptase and guided by a prime-editing guide RNA (pegRNA). The latter guides the system to the targeted DNA site and encodes the desired correction [44]. Further future work needs to be carried out in order to establish the potential and off-target edits of this new technology.

## Figures and Tables

**Table 1:** Different strategies used to edit the genome using the CRISPR/Cas9 tool.

	Plasmid-Borne CRISPR/Cas9 System	Cas9/sgRNA Complex	Cas9 mRNA and sgRNA
**Principle**	A plasmid is encoding the designed sgRNA and Cas9 protein under the appropriate promoters	Delivery of sgRNA complexed with Cas9 protein	Delivery of sgRNA and Cas9 mRNA
**Advantages**	Both Cas9 protein and sgRNA are carried on the same vector; ensures that both are expressed in the same cell. This system offers improved stability, especially during handling and manufacturing, compared to the other two strategies. High versatility and customizability as multiple sgRNAs can be cloned in the same plasmid. The plasmid can also contain the homology directed repair (HDR) template. A fluorescent protein can also be included in the plasmid to label cells expressing the Cas9 enzyme. Relatively low cost. Good reproducibility.	Simplicity of the system’s preparation, as Cas9 protein spontaneously forms a complex with sgRNA being oppositely charged. The effect of the complex is the fastest among the 3 strategies as neither transcription nor translation to Cas9 protein are needed. Minimal off-target effects and cell toxicity.	This approach works faster than the plasmid strategy towards editing the targeted gene, as only the translation of Cas9 mRNA is required to produce the Cas9 protein. Fewer off-target effects compared to the plasmid-based system. Lower cell toxicity.
**Disadvantages**	Low transfection efficiency of primary cells. Potential for the random insertion of plasmid fragments into the gene. Cytotoxicity associated with the use of DNA and of bacterial DNA sequences present in the plasmid.	The intracellular delivery of the Cas9 protein is very challenging especially because of its large size (about 160 kDa). Purification of the Cas9 protein, free from endotoxin contamination, is expensive.	Instability of RNA
**References**	[25,26]	[27,28]	[29]

**Table 2:** Overview of studies involving CRISPR/Cas9 technology in Alzheimer’s disease treatment.

Targeted Gene	Delivery System	FAD or SAD	Cell Lines Tested	Animals Tested	Ref.
*KM670/671NL **APP*** (**APP^swe^**) mutation	CRISPR/Cas9 delivered via recombinant adeno-associated virus (rAAV)	FAD	- Human APP^swe^ fibroblasts - Primary neuronal cells from *Tg2576* mice embryos	Intra-hippocampal injection in *Tg2576* mouse model	[25]
**BACE1**	Nanocomplexes composed of R7L10 peptide complexed with Cas9-sgRNA ribonucleoprotein	FAD	- Primary cultured neurons from mice embryos - Human embryonic stem cells and human induced pluripotent stem cells - GFP^+^ HEK293T cells	Intrahippocampal injection in: - *5xFAD* transgenic mice co-expressing 5 familial AD mutations - *App* knock-in transgenic mice	[27]
***APP*** at the extreme C-terminus	Lentiviral CRISPR/Cas9 system	FAD	- HEK293 and neuro2a cells	Injection into the dentate gyrus of *C57BL/6* mice	[30]
***APOE* ε4** (converting it into *APOE3r*)	CRISPR/Cas9 plasmids combined with cytidine deaminase enzyme (to maintain sgRNA programmability while avoiding double-stranded DNA breaks)	SAD	- HEK293T cells - Immortalized mouse astrocytes containing the *APOE* ε4 isoform of the *APOE* gene	-	[31]
***APOE* ε4**	Lentiviral CRISPR/Cas9 system	SAD	- Mouse astrocytic cells expressing the human *APOε3* or *APOε4* gene	-	[32]
